# MetaProFi: an ultrafast chunked Bloom filter for storing and querying protein and nucleotide sequence data for accurate identification of functionally relevant genetic variants

**DOI:** 10.1093/bioinformatics/btad101

**Published:** 2023-02-24

**Authors:** Sanjay K Srikakulam, Sebastian Keller, Fawaz Dabbaghie, Robert Bals, Olga V Kalinina

**Affiliations:** Helmholtz Institute for Pharmaceutical Research Saarland (HIPS), Helmholtz Centre for Infection Research (HZI), 66123 Saarbrücken, Germany; Graduate School of Computer Science, Saarland University, 66123 Saarbrücken, Germany; Interdisciplinary Graduate School of Natural Product Research, Saarland University, 66123 Saarbrücken, Germany; Helmholtz Institute for Pharmaceutical Research Saarland (HIPS), Helmholtz Centre for Infection Research (HZI), 66123 Saarbrücken, Germany; Graduate School of Computer Science, Saarland University, 66123 Saarbrücken, Germany; Helmholtz Institute for Pharmaceutical Research Saarland (HIPS), Helmholtz Centre for Infection Research (HZI), 66123 Saarbrücken, Germany; Institute for Medical Biometry and Bioinformatics, Heinrich Heine University Düsseldorf, Medical Faculty, 40225 Düsseldorf, Germany; Center for Digital Medicine, Heinrich Heine University, 40225 Düsseldorf, Germany; Department of Internal Medicine V—Pulmonology, Allergology, Intensive Care Medicine, 66421 Homburg, Germany; Helmholtz Institute for Pharmaceutical Research Saarland (HIPS), Helmholtz Centre for Infection Research (HZI), 66123 Saarbrücken, Germany; Drug Bioinformatics, Medical Faculty, Saarland University, 66421 Homburg, Germany; Center for Bioinformatics, Saarland University, 66123 Saarbrücken, Germany

## Abstract

**Motivation:**

Bloom filters are a popular data structure that allows rapid searches in large sequence datasets. So far, all tools work with nucleotide sequences; however, protein sequences are conserved over longer evolutionary distances, and only mutations on the protein level may have any functional significance.

**Results:**

We present MetaProFi, a Bloom filter-based tool that, for the first time, offers the functionality to build indexes of amino acid sequences and query them with both amino acid and nucleotide sequences, thus bringing sequence comparison to the biologically relevant protein level. MetaProFi implements additional efficient engineering solutions, such as a shared memory system, chunked data storage and efficient compression. In addition to its conceptual novelty, MetaProFi demonstrates state-of-the-art performance and excellent memory consumption-to-speed ratio when applied to various large datasets.

**Availability and implementation:**

Source code in Python is available at https://github.com/kalininalab/metaprofi.

## 1 Introduction

With the technological advancement in the field of next-generation sequencing (NGS) over the past decade, there is a rapid growth in the amount of available biological sequencing data in public databases e.g. European Nucleotide Archive (ENA) (Leinonen *et al.*, 2011a), and Sequence Read Archive (SRA) (Leinonen *et al.*, 2011b). NGS data have become an invaluable resource in various fields of life science research. As the size of the databases reached the petabyte scale, it has become difficult to support online searches in these databases. As great power comes with great responsibility, the requirement for tools to process, store and query large collections of sequence data without high memory and storage requirements constitutes a computational challenge. Analyzing these abundant data will lead to opportunities for great scientific discoveries.

To reduce the required time and memory, a probabilistic data structure can be used to summarize and deliver fast approximate answers. Probabilistic membership data structures aid in determining whether an item/element is present or not. Several such data structures are already available, e.g. Bloom filter, Cuckoo filter, Quotient filter, etc. These data structures differ in their ability to offer approximation or definite answers. Many of these data structures are predominantly used in streaming applications and database lookups before performing any expensive operations. These data structures guarantee zero false negatives, thus if such a data structure returns an answer of a non-existent key, then the database fetch/read is not required, saving time.

In the last few years, several tools utilizing Bloom filter data structures for storing and querying large sequence datasets became available: SBT (Solomon and Kingsford, 2016), HowDeSBT (Harris and Medvedev, 2020), BIGSI (Bradley *et al.*, 2019), COBS (Bingmann *et al.*, 2019), DREAM-Yara (Dadi *et al.*, 2018), Rambo (Gupta *et al.*, 2021), kmtricks (Lemane *et al.*, 2021) and others. Other tools utilize other variants of probabilistic data structures: Squeakr (Pandey *et al.*, 2018a) in combination with Mantis (Pandey *et al.*, 2018b), BCALM2 (Chikhi *et al.*, 2016) in combination with REINDEER (Marchet *et al.*, 2020). Custom indexes are built either of the raw sequencing data or of the data from curated databases and later allow the sequences of interest to be queried against these custom indexes to find which samples in the index contain the query sequence. Although several tools are available, none of them supports amino acid sequence indexing, creating a major gap in the field. MetaProFi bridges this gap by proposing the first data structure that can not only store both amino acid and nucleotide sequence data but also allows for querying amino acid-based indices with nucleotide queries, internally performing a six-frame translation of the query. A better conservation of protein sequence makes MetaProFi more robust in detecting non-identical, but closely related sequences, which, in combination with the possibility of querying for non-perfectly identical sequences, ensures its larger flexibility.

**Fig. 1.**
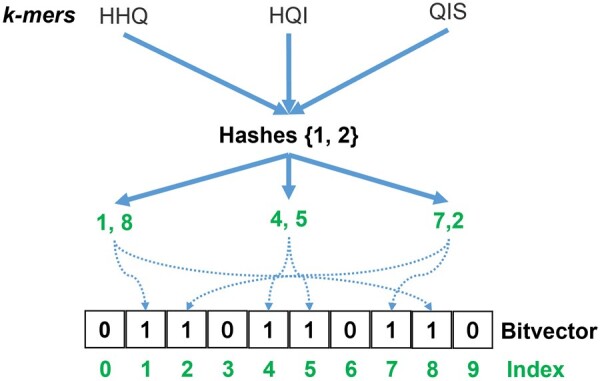
Bloom filter data structure: two hash functions are applied to each of the three strings and the return values of hashing are used as an index in the bit vector to flip the zero in the corresponding index position to one

To this end, we introduce MetaProFi, a first-of-its-kind tool for indexing amino acid sequences that also supports nucleotide sequence indexing, using Bloom filters as the underlying data structure. Bloom filter (Fig. 1) is a probabilistic set-membership data structure that stores the presence or absence of items/elements in a bit vector and can be queried for presence or absence. Bloom filters guarantee zero false negatives. The bit vector is filled with binary values where zero indicates absence and one indicates presence: we start by filling the bit vector with zeros; for each given string, we apply h hash functions and use the return value of hashing as an index in the bit vector to flip the zero in the corresponding index position to one. Collisions (returning the same hash value for different strings) can be avoided by using large Bloom filters and perfect hash functions, but in a realistic setting, this is not possible, thus false positives arise. Their number can be reduced by increasing the size of the Bloom filter and manipulating the number of hash functions used.

MetaProFi combines the power of a variant of Bloom filter data structure which we call packed Bloom filter (see Section 2) with data chunking and compression to construct the Bloom filter matrix efficiently to index all the observed *k*-mers (presence/absence) for fast queries with reduced memory, storage and runtime requirements.

The novel features of MetaProFi include (i) indexing support for both nucleotide and amino acid sequences; (ii) a possibility for querying amino acid sequence index using nucleotide sequences; (iii) a seamless update of previously built indexes with new data/samples; (iv) considerable storage reduction compared to the state-of-the-art tools.

MetaProFi serves various applications: one can index all the sequences available in UniProtKB (The UniProt Consortium, 2021) in a protein-based index. One of the possible applications of such an index would be a fast alignment-free sequence search that can be used, for example, to find resistance-associated genes directly from metagenomics data. MetaProFi allows exact query search expecting every *k*-mer in the query sequence to be present, and also supports approximate search using a threshold (*T*) when only the fraction of *k*-mers larger than *T* have to be found. As a proof of concept, we have constructed a MetaProFi index for UniProtKB bacterial sequences (amino acids) on two different levels (organism level and sequence level), for the Tara Oceans (Karsenti *et al.*, 2011) dataset containing nucleotide metagenomic sequencing data collected across all oceans. Additionally, we used the dataset described in Harris and Medvedev (2020) which consists of 2585 human RNA-seq experiment results comprising blood, brain and breast samples to demonstrate the storage, memory, run time, scalability and query performance.

## 2 Materials and methods

MetaProFi is developed in Python and is parallelized to take advantage of all the available CPU cores on today's modern computing systems in order to achieve the best performance. MetaProFi accepts both FASTA and FASTQ formats and uses pyfastx (Du *et al.*, 2021) Python library for parsing the files efficiently.

### 2.1 Construction of chunked Bloom filter matrix

MetaProFi builds Bloom filters in the form of a matrix directly, unlike other tools that construct individual Bloom filters for each sample first and then construct a matrix (and/or other forms) for indexing. The rows in the matrix represent hash indexes while each column represents a Bloom filter of length *m* of a sample. Since we cannot construct large matrices in memory, MetaProFi splits the number of input samples (*Y*) into *N* batches of small samples. *N* is estimated based on the Bloom filter size *m*, the number of samples in the input, and a user-defined maximum memory usage threshold.

In the matrix, MetaProFi uses an 8-bit unsigned integer (UINT8) data type to store bits for eight *k*-mers by applying bit manipulations to a UINT8 integer (a packed Bloom filter). Once all *k*-mers in a batch of samples are hashed and the respective bits are flipped in the Bloom filter matrix, MetaProFi applies data chunking using the Zarr library (Miles *et al.*, 2022) and compression (Zstandard algorithm, https://github.com/facebook/zstd) techniques to reduce the storage requirements. This means that the Bloom filters from all samples of the batch are chunked into *C* pieces that are compressed and written to the hard drive. *C* is calculated in such a way that the portion of the full Bloom filter matrix corresponding to *m*/*C k*-mers in all *Y* samples can be loaded to memory.

By applying compression on each chunk, we achieve a remarkably better compression ratio (Table 2) than when compressing individual Bloom filters and through this, we offer a significant storage reduction. This process is repeated for all *N* sample batches. Using the Zarr library to store the chunks on disk ensures that the chunks from all samples that correspond to the same set of Bloom filter rows are stored in such a way that they can be assessed and loaded to memory simultaneously, and the whole bit vector corresponding to a single hash value in all *Y* samples can be extracted.

MetaProFi utilizes POSIX shared memory as the matrix backend, and this enables MetaProFi to efficiently access the matrix through multiple processes. Using POSIX shared memory allows us a zero-copy data transfer between multiple processes, at the same time achieving a significant reduction in the Bloom filter construction time.

### 2.2 Index construction for the full Bloom filter matrix

**Fig. 2:**
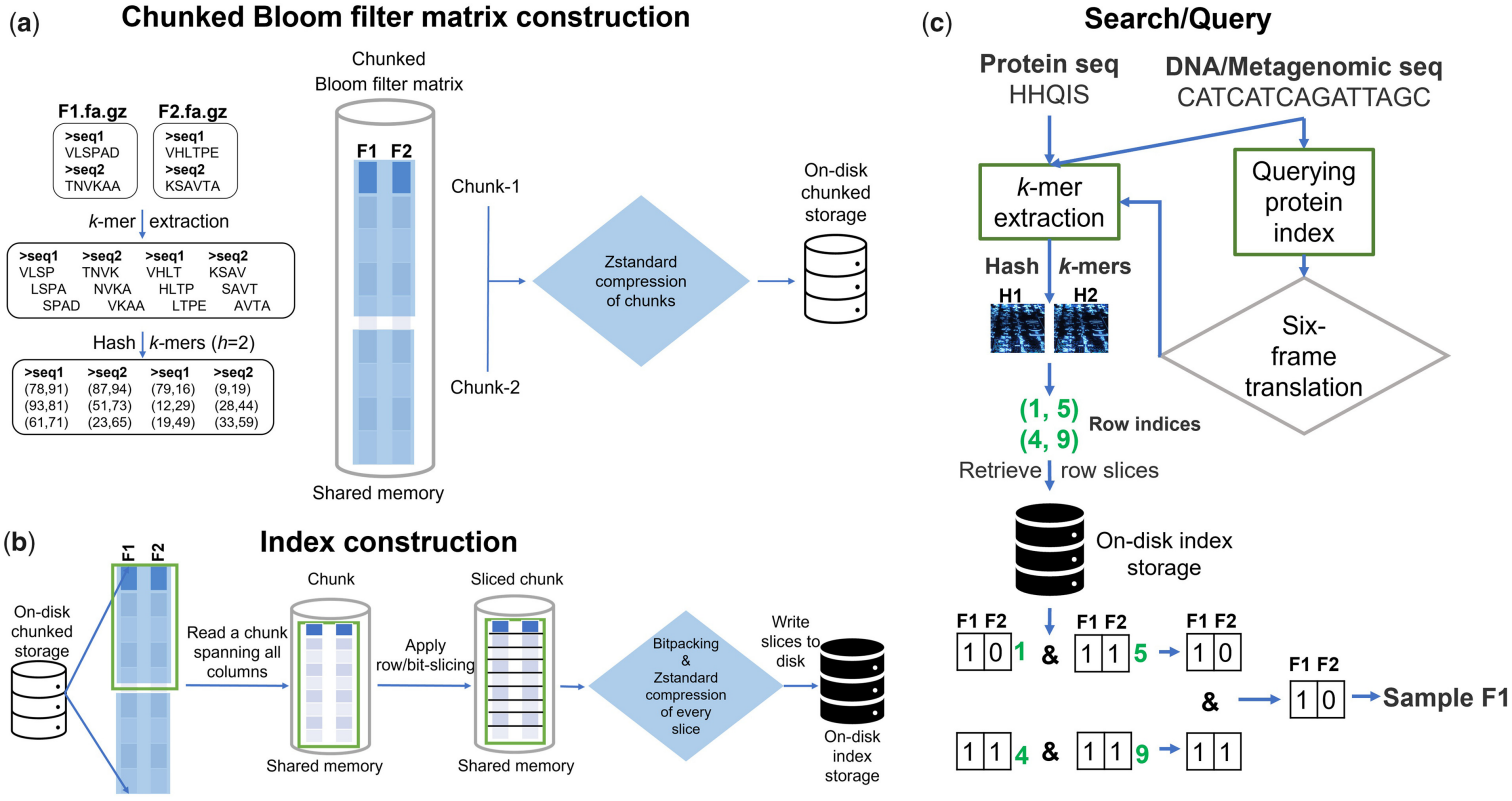
Overview of MetaProFi pipeline: (**a**) Chunked Bloom filter matrix construction, (**b**) Index construction and (**c**) Search/query pipeline

Since MetaProFi’s Bloom filter matrix is stored in batches and chunks, direct queries for a large number of *k*-mers need to enumerate all chunks and likely will be slow. So, we build a dedicated efficient index structure (Fig. 2b) for the Bloom filter matrix such that querying every *k*-mer has a constant time cost (Bradley *et al.*, 2019) independent of the number of batches.

The indexing data structure is an array of size *m* (size of the Bloom filter), where each cell in the array corresponds to a row [or, equivalently, a bit-slice (Bradley *et al.*, 2019)] across all samples (columns) from the Bloom filter matrix. To construct this index MetaProFi re-creates a POSIX shared memory matrix of size *X *×* Y*, where *Y* is equal to the total number of samples in the Bloom filter matrix and *X *=* m*/*C* is the number of rows that can be read into the memory without crossing the maximum memory limit set in the configuration file. MetaProFi reads *X* rows from each chunk spanning all *N* sample batches, on the fly unpacks the UINT8 packed Bloom filter to individual bits and writes the several unpacked bits of *X* rows and *Y*/*N* samples corresponding to a batch to the shared memory matrix in parallel. Once again to benefit from data chunking and compression, MetaProFi applies this technique to these *X* rows and distributes the compressed chunks to multiple processes which are then written to the disk. MetaProFi repeats the procedure until all rows from the Bloom filter matrix are indexed. MetaProFi also supports updates to the index for adding new samples.

### 2.3 FAQIndexing for FASTA/FASTQ files

MetaProFi can build the Bloom filter matrix either using a collection of FASTA/FASTQ files where each file is a single sample or using a single FASTA/FASTQ file where every sequence is treated as a sample. In the latter case, to accelerate the construction of the MetaProFi Bloom filter matrix, we introduce a dedicated indexing data structure. We refer to this as FAQIndex (FASTA/FASTQ index) hereafter. Inspired by pyfastx (Du *et al.*, 2021), we have implemented an LMDB (Lightning Memory-Mapped Database) (https://lmdb.readthedocs.io/en/release/) based indexing for FASTA/FASTQ (compressed and uncompressed) files. We used LMDB as a storage backend for its fast querying in addition to the capability to efficiently share the same database with multiple processes. The FAQIndex contains six columns: the sequence number, name of the sequence, sequence start offset, byte length of the sequence and the number of bases in the sequence. This FAQIndex requires less storage compared to pyfastx’s index as we do not store additional information, such as sequence type and read parameters. Every row in our FAQIndex is serialized and then compressed for reducing the storage requirements.

### 2.4 Querying/searching the MetaProFi index

MetaProFi accepts both raw sequence and FASTA/FASTQ files as inputs for querying the index (Fig. 2c). When a multi-sequence file is used for querying, MetaProFi automatically constructs a small FAQIndex of the file for distributing the query sequences to multiple cores/processes. MetaProFi collects the hashes of each *k*-mer from every sequence in parallel processes. The MetaProFi index is queried with these hash values and bit-slices corresponding to each *k*-mer are retrieved.

MetaProFi allows exact query search where every *k*-mer in the query sequence is expected to be present in a sample and also supports approximate search using a threshold (*T*) when only a fraction of *k*-mers larger than *T* have to be found. In addition to querying with an amino acid sequence against an index built using amino acid samples and a nucleotide sequence against a nucleotide index, MetaProFi allows querying an amino acid index using nucleotide sequences (e.g. metagenomic reads, contigs or assembled genomes) directly. To this end, MetaProFi performs a six-frame translation of the nucleotide sequences and uses all six translated sequences as queries to search the amino acid index. This is not expected to create any false-positive hits, since in the five non-biological frames a stop codon is expected to occur approximately every 21 codons, and hence not more than 10 consecutive *k*-mers can be matched in a spurious translation frame.

### 2.5 False-positive rate

Bloom filters belong to the class of probabilistic data structures with a zero false-negative rate, and they are prone to false positives by design. However, the false-positive rate of the Bloom filters can be controlled by increasing the size of the Bloom filter and the number of hash functions used. As discussed in Bradley *et al.* (2019), one can also calculate the false-positive rate of the query, which depends on (i) the number of samples in a dataset, (ii) the size of the *k*-mer, (iii) the maximum number of acceptable false discoveries per query and (iv) the shortest length of the query sequence to be supported. Using these parameters, a false-positive rate per query can be calculated. The exact formulation for the false-positive rate calculation is presented, for example, in Bradley *et al.* (2019).

### 2.6 Computing setup

Performance evaluations were done on a Dell server with the following configuration: AMD EPYC 7702 2.0 GHz CPU with 1.5 TB RAM, Intel SSD DC P4610 3.2 TB (2.9 TiB) and CentOS 7 (kernel 3.10.0-1160.24.1.el7.x86_64) operating system. MetaProFi was allocated with 64 cores, and 60 GiB RAM for all its experiments, and the same for other tools wherever possible during benchmarking. All input files were stored on a non-RAID NVMe NFS file system, and outputs were stored on the Intel SSD DC P4610 3.2 TB disk.

### 2.7 UniProtKB dataset indexing

Two types of MetaProFi indexes (one at the organism level and one at the sequence level) were constructed for all bacterial sequences in the UniProtKB (Swiss-Prot and TrEMBL) database downloaded in July 2021 with a total size of 64 GiB. The entire Swiss-Prot and TrEMBL datasets were downloaded from UniProt’s FTP site and the accession ids for all the bacterial sequences were downloaded by performing a search with ‘taxonomy: bacteria’ on UniProt’s search interface.

Three parameters define the architecture of Bloom filters in MetaProFi: *m*, *h* and *k*, where *m* is Bloom filter size, *h* is the number of hash functions to be applied on every *k*-mer and *k* is the size of the *k*-mer. In MetaProFi, for organism-level indexing, we used the following parameters: *m *=* *600 000 000; *h *=* *2; *k *=* *11. With these parameters, the false-positive rate was 0.47, while the false-positive rate per query is 10^−6^ if the query sequence size is of a minimum of 35 characters. For sequence-level indexing of the UniProtKB bacterial dataset, we used the same Bloom filter parameters with one change, *m *=* *600 000; and with these parameters, the false-positive rate was 0.0156 and the per query false-positive rate is 10^−6^ if the query sequence size is of a minimum of 34 characters.

### 2.8 Tara Oceans dataset indexing

MetaProFi is mainly developed to fill the technology gap of amino acid sequence indexing, but for benchmarking purposes, nucleotide sequence indexing support is also added. With this feature, we downloaded 4 TiB of compressed Tara Oceans dataset (study accession: PRJEB1787) from the ENA archive consisting of 249 samples containing 495 FASTQ files. For MetaProFi indexing of the Tara Oceans dataset, we used the following Bloom filter parameters: *m *=* *40 000 000 000; *h *=* *1; *k *=* *31. With these parameters, the false-positives rate was 0.3782 and the false-positive rate per query *k*-mer is 10^−6^ if the query sequence size is of a minimum of 50 characters. Since we wanted to benchmark MetaProFi’s performance with other tools which do not provide an option to change the number of hash functions used, we set MetaProFi to use only one hash function.

We compared MetaProFi’s performance with kmtricks (v1.1.1), and kmtricks was run using the same Bloom filter parameters as above and without filtering *k*-mers that appear only once. Further, kmtricks was run in the ‘hash: bft: bin’ mode which only performs the Bloom filter matrix construction instead of the *k*-mer counting. We chose other parameters (number of cores, *k*-mer length) to match those of MetaProFi for a fair comparison. For kmtricks in combination with HowDeSBT, we used only 1% of bits to be considered from all Bloom filters during clustering and indexing, since the value recommended by the HowDeSBT tutorial (https://github.com/medvedevgroup/HowDeSBT/tree/master/tutorial) lead to prohibitively long runtimes.

For evaluating query performance, we randomly selected 1000 reads from the 495 FASTQ files of the Tara Oceans dataset and used them for querying.

### 2.9 Human RNA-seq dataset indexing

For benchmarking, we also used a human RNA-seq dataset consisting of 2585 samples (2.7 TiB) that were also used in Harris and Medvedev (2020). We downloaded this dataset from the SRA using the parallel-fastq-dump (https://github.com/rvalieris/parallel-fastq-dump) tool, accession numbers were obtained from Harris and Medvedev (2020).

We divided this experiment into two sets. First, a subset of 650 samples (referred to as RNA-seq-mini hereafter) was randomly selected for building a small index to compare the performance of several tools: HowDeSBT (v2.00.02 20191014), kmtricks (v1.1.1), COBS (v0.1.2), Squeakr (v1.0) in combination with Mantis (v0.2.0) and MetaProFi. Second, all 2585 samples (referred to as RNA-seq hereafter) were indexed using both COBS (v0.1.2) and MetaProFi.

For the RNA-seq-mini dataset, we chose the following Bloom filter parameters: *m *=* *2 000 000 000; *h *=* *1; *k *=* *21. With these parameters, the false-positive rate was 0.09 and the false-positive rate per query is 10^−5^ with a minimum query size of 31 characters. To level the comparison and consistency between all tools, we first constructed compacted De Bruijn Graphs (DBGs) for all 650 samples using BCALM2 (v2.2.3) (Chikhi *et al.*, 2016), while removing all *k*-mers that appear only once and then used this as input to all the tools for the benchmark. For Mantis, we built Squeakr input files and also made sure to remove all *k*-mers that appear only once. It must be noted that MetaProFi does not require these pre-processing steps. During the execution of all tools, we made sure that none of them repeats the removal of *k*-mers that appear only once step. Also, we ran kmtricks in the ‘hash: bft: bin’ mode which will only perform the Bloom filter matrix construction instead of the *k*-mer counting. We chose other parameters (number of cores, *k*-mer length) to match those of MetaProFi for a fair comparison. For HowDeSBT and kmtricks in combination with HowDeSBT, we used only 1% of bits to be considered from all Bloom filters during clustering and indexing, since the value recommended by the HowDeSBT tutorial (https://github.com/medvedevgroup/HowDeSBT/tree/master/tutorial) lead to prohibitively long runtimes.

For the RNA-seq dataset, we obtained the Bloom filter parameters from (Marchet *et al.*, 2020). Bloom filter parameters were the following *m *=* *2 000 000 000; *h *=* *1; *k *=* *21. This dataset was used as it is without applying any *k*-mer filtering.

For evaluating query performance, we downloaded a FASTA file comprising 70 866 transcripts, following (Marchet *et al.*, 2020). We then extracted the first 1000 transcripts using pyfastx (Du *et al.*, 2021) and used it for querying both RNA-seq and RNA-seq-mini indexes of all tools. RAM utilization was monitored through the Linux command-line utility atop (via the command atop -mp).

## 3 Results

MetaProFi allows the indexing of large numbers of samples/datasets. MetaProFi (Fig. 2) combines the power of a probabilistic data structure with data chunking and compression to store large Bloom filters and to create indexes for fast querying. The key methodological novelty of MetaProfi is its ability to index amino acid sequences and enable the querying of amino acid sequence index using nucleotide sequences. This allows disregarding synonymous mutations and focusing directly on sequence variants that impact the protein sequence and hence may impact the corresponding protein functions. Moreover, more efficient exact sequence searches are possible also for non-exactly matching strains that contain only silent mutations. Additionally, since protein sequence homology is detectable across longer evolutionary distances, homologous sequences can be detected on the level where nucleotide-level similarity fails.

### 3.1 MetaProFi performance

To evaluate the performance of MetaProFi, we used UniProtKB, Tara Oceans and human RNA-seq and RNA-seq-mini datasets (see Section 2 for details) for the index construction. For UniProtKB, two types of indexes were created: one at the organism level and the other at the sequence level. Since no other tool is available to perform amino acid *k*-mer indexing we added support for nucleotide indexing to MetaProFi to enable comparison against other tools and we indexed the Tara Oceans and the RNA-seq datasets for benchmarking purposes.

#### 3.1.1 UniProtKB dataset indexing

For UniProtKB organism-level indexing, the dataset was constructed by extracting individual bacterial sequences using their accession ids with criteria on the minimum length of the sequence (*k *=* *11) and then grouping them by their organism’s name (OS field value in the fasta header) to obtain 100 384 uncompressed individual fasta files containing a total of 46 511 863,142 *k*-mers. MetaProFi constructs the Bloom filter matrix in 38.05 min and the index in 971 min using under 60 GiB of RAM and under 135 GiB of disk space (Table 1). Storage size is directly proportional to the size of the Bloom filter and the number of samples in the dataset: if MetaProFi had used a regular Bloom filter, it would require 6.85 TiB (size of the Bloom filter times number of fasta files/samples, i.e. 600 000 000 × 100 384 = 60 230 400 000 000 bits, which is equal to 6.85 TiB) disk space for storing the uncompressed Bloom filter matrix. With MetaProFi’s optimizations and techniques, we provide a 50-fold compression and can construct Bloom filter matrices for a large number of datasets or use very large Bloom filters that have a low false-positive rate, while still storing them efficiently.

**Table 1.** MetaProFi indexing results for UniProtKB bacterial dataset

	Time (min)	RAM (GiB)	CPU cores	Disk (GiB)
Organism level				
Bloom filter matrix	38.05	<60	64	139
MetaProFi index	971	<60	64	135
Sequence level				
Bloom filter matrix	65.38	<60	64	232
MetaProFi index	1357	<60	64	210

To demonstrate MetaProFi’s scalability we used all the bacterial sequences that were extracted from the UniProtKB dataset, 334 984 sequences from Swiss-Prot and 151 450 171 sequences from TrEMBL. We monitored the construction of the FAQIndex (see Section 2.3) for the compressed UniProtKB bacterial input dataset of size 32 GiB during the Bloom filter matrix build step. MetaProFi’s FAQIndexing tool took 17 min to construct the FAQIndex and used 11 GiB of storage and a maximum of 1 GiB RAM. Using LMDB as the underlying database reduces the RAM consumption, as we do not retain data in memory and write the data for every sequence to the disk as soon as they are populated. Using the FAQIndex of the UniProtKB bacterial input dataset (151 785 155 sequences), we created a sequence-level MetaProFi index. The Bloom filter matrix and index construction time are comparable with the organism-level case. The disk requirements are twice as large, and RAM consumption is comparable (Table 1). These results show that MetaProFi is scalable to hundreds of millions of samples.

#### 3.1.2 RNA-seq-mini dataset indexing

We created a subset of 650 samples out of 2585 samples of the RNA-seq dataset to benchmark MetaProFi’s performance with other tools such as HowDeSBT, kmtricks in combination with HowDeSBT, COBS and Squeakr in combination with Mantis. We first built the compacted DBGs for all 650 samples (while removing *k*-mers that were present only once) and then used them as the input (see Section 2 for details). From the results (Table 2), we can see that COBS has the best total runtime followed by MetaProFi, whereas the total disk consumption of MetaProFi is the smallest.

**Table 2.** RNA-seq-mini dataset benchmark comparisons

Tool	RAM (GiB)	CPU cores	Disk BF (GiB)	Disk index (GiB)	Disk total (GiB)	Time BF (min)	Time index (min)	Total time (min)
HowDeSBT	**2.4**	64	152	**4.4**	168.4	51.94	93.1	145.1
kmtricks + HowDeSBT	286.39 + 4	64	156	4.4	308 + 12	54.49	383.9	438.39
MetaProFi	12	64	**8.2**	9.4	**17.6**	**4.37**	20.42	24.79
COBS	12	64	–	51	54	–	**8.59**	**8.59**
Squeakr + MANTIS	7.3 + 49	64	28	14	42	145.22	33.27	178.49

BF, Bloom filter; Disk total, total storage used for BF, index and intermediate files; Time total, total time for constructing BF and index; –, N/A. Bold face indicates the best performing tool.

We also benchmarked the query performance of all these tools, for which we downloaded a fasta file from RefSeq (O'Leary *et al.*, 2016) comprising 70 866 human transcripts from RefSeq, as reported in Marchet *et al.* (2020). We extracted the first 1000 transcripts and used them for querying. The results show that HowDeSBT is faster when performing exact querying (*T *=* *100%) (exact), whereas MetaProFi is equally fast in both exact and approximate (*T *=* *75%) searches and requires a very low amount of memory (Table 3).

**Table 3.** Query performance benchmark of 1000 transcripts

Tool	RAM (GiB)	CPU cores	Time (s) (*T* = 100%)	Time (s) (*T* = 75%)
HowDeSBT	**0.61**	–	**22**	558
kmtricks + HowDeSBT	0.64	20	2952	2957
MetaProFi	1.9	20	29	**33**
COBS	25.1	20	234	228
Squeakr + MANTIS	14	–	37	–

Bold face indicates the best performing tool.

#### 3.1.3 RNA-seq dataset indexing

We built a full index with all the samples (2585 samples; 6 432 932 578 661 *k*-mers, *k *=* *21) from the human RNA-seq experiments obtained from the SRA (Leinonen *et al.*, 2011b) using MetaProFi and COBS. MetaProFi takes about 20% longer time than COBS and requires around 30% less storage (Table 4). We did not attempt to build the index with other tools as they were found to be prohibitively slow for the small RNA-seq-mini dataset.

**Table 4.** RNA-seq dataset benchmark comparisons

Tool	RAM (GiB)	CPU cores	Disk BF (GiB)	Disk index (GiB)	Disk total (GiB)	Time BF (min)	Time index (min)	Total time (min)
MetaProFi	**59**	64	295	**333**	**628**	1108	**127**	1235
COBS	69.4	64	N/A	935	996	N/A	1000	**1000**

Bold face indicates the best performing tool.

We then used this index for querying 1000 transcripts (Table 5). MetaProFi was 6–7 times faster than COBS and required much less memory as well.

**Table 5.** Query performance benchmark of 1000 transcripts

Tool	RAM (GiB)	CPU cores	Time (s) (*T* = 100)	Time (s) (*T* = 75)
MetaProFi	**3.4**	64	**43**	**48**
COBS	92.5	64	290	290

Bold face indicates the best performing tool.

#### 3.1.4 Tara Oceans dataset indexing

To compare MetaProFi with the state-of-the-art tools, we used kmtricks (Lemane *et al.*, 2021), a *k*-mer counting tool that allows building Bloom filters that can be used for constructing a *k*-mer index using a variant of HowDeSBT (Harris and Medvedev, 2020) implemented in its package. We applied both tools to the Tara Oceans dataset which contains a total of 3 431 551 187 218 *k*-mers (*k *=* *31).

During Bloom filter construction kmtricks was 2–3 times faster than MetaProFi while consuming 2–3 times more storage than MetaProFi (Table 6). After 120 h the kmtricks + HowDeSBT index construction (1% of bits were considered from each filter) was terminated, and we report only the numbers we observed up until the termination without any extrapolation (we assume that the computation might have taken several more days as only less than half of the Bloom filters were indexed). On the other hand, we can see that MetaProFi required only 4.65 h to build an index. This shows that MetaProFi can index datasets containing trillions of *k*-mers in a reasonable amount of time yet only requiring low amounts of memory and storage.

**Table 6.** Tara Oceans dataset benchmark comparisons of MetaProFi and kmtricks

Tool	RAM (GiB)	CPU cores	Disk BF (GiB)	Disk index (GiB)	Disk total (GiB)	Time BF (min)	Time index (min)	Time total (min)
MetaProFi	68	64	**643**	750	**1393**	2642	279	2921
kmtricks + HowDeSBT	**47**	64	1228.8	>390^a^	2344 + 390^a^	**865**	>7217^a^	>8082^a^

BF, Bloom filter; disk total, total storage used for BF, index and intermediate files; Time total, total time used to construct BF and index.

Bold face indicates the best performing tool.

a Terminated after 120 h, data reported as it is at the time of termination.

To demonstrate MetaProFi’s query performance, we randomly selected 1000 reads from the 495 FASTQ files of the Tara Oceans dataset used for constructing the index. These 1000 reads were queried against the Tara Oceans MetaProFi index using exact search (*T *=* *100%) and approximate search (*T *=* *75%). The query run times were 164 s for the exact search (*T *=* *100%) and 166 s for the approximate search (*T *=* *75%). We observed a peak memory usage of 14.3 GiB. We were unable to compare the query results with the kmtricks + HowDeSBT setup as the index construction had to be terminated after 120 h.

From our benchmarking results, we can see that MetaProFi reduces the amount of disk usage even for very large Bloom filters, compared to the state-of-the-art tools, constructs index in little time, and performs better during querying.

## 4 Discussion

MetaProFi for the first time presents a possibility to index directly protein sequences, which makes calling variants in coding sequences a much easier task. In addition, it comprises a mode of usage centered around nucleotide sequences, which makes it possible to compare it to other tools in the field. In these comparisons, MetaProFi demonstrated state-of-the-art performance with the best runtime/memory ratio. MetaProFi was able to build *k*-mer indexes rapidly for multiple datasets of different sizes demonstrating it can scale in any direction.

Nevertheless, the most important feature of MetaProFi, which makes it stand out among other tools, is that it can build indexes for amino acid sequences and enables querying of an amino acid index using nucleotide sequences. This approach was taken with an intended goal in mind: to most efficiently store and query sequence data coming from metagenome samples, primarily bacterial metagenomes. Storing protein data makes the search more flexible and offers many advantages, while the only disadvantage is that the information in non-coding regions is lost. In this scenario, we consider this a little loss, since bacterial genomes contain comparatively little non-coding sequence, and a lot of important markers are detected at the protein level, e.g. markers of antibiotic resistance. Potential advantages include, for example, the possibility to conduct very fast and efficient searches with the *k*-mer presence threshold *T *=* *100% for closely related, but not identical DNA sequences that contain no missense mutations at the protein level. On the other hand, remote homologs can be detected in cases, when the sequence similarity on the DNA level drops but is still detectable at the protein level.

While other state-of-the-art tools do not typically offer the functionality to store amino acid-based indexes, the change of the code to allow it would not be a large one *per se*, although would require numerous adjustments. However, the option to query an amino acid-based index with a nucleotide query is a non-trivial one and is unique to MetaProFi.

To conclude, we developed MetaProFi, a first-of-its-kind amino acid *k*-mer indexing tool with added support for indexing nucleotide sequences that efficiently builds indexes from tens of samples to hundreds of millions of samples with reduced memory, storage and runtime than its predecessors. Through our proof-of-concept index construction for multiple datasets, we demonstrated that we could grow our index horizontally (samples) or vertically (Bloom filter size), and yet it requires very little storage and memory without compromising the performance both for index building and querying tasks. MetaProFi can be further developed to support distributed computing infrastructure in addition to the current single system-specific deployment setup.
